# Frederick Graham Lilley

**Published:** 1932

**Authors:** 


### FREDERICK GRAHAM LILLEY, L.D.S., R.C.S.

WITHIN a short time of his appointment to the office of Dental House Surgeon at the Bristol General Hospital, Mr. F. G. Lilley was attacked by a virulent form of pneumonia, and died on 30th September after an illness lasting two days.

Both as a student and during his short tenure of office Mr. Lilley showed exceptional promise, combining sound professional knowledge with a charming personality. He was the only son of Dr. and Mrs. Frederick Lilley, of Weston-super-Mare.

